# Performance Enhancement of Ce_0.8_Sm_0.2_O_1.9_-Supported SOFC by Electrophoretic Formation of Modifying BaCe_0.8_Sm_0.2_O_3_ and Ce_0.8_Sm_0.1_Pr_0.1_O_1.9_ Layers

**DOI:** 10.3390/membranes13050484

**Published:** 2023-04-29

**Authors:** Elena Pikalova, Elena Kalinina

**Affiliations:** 1 Laboratory of Solid Oxide Fuel Cells, Institute of High Temperature Electrochemistry, Ural Branch of the Russian Academy of Sciences, Yekaterinburg 620137, Russia; e.pikalova@list.ru; 2 Department of Environmental Economics, Graduate School of Economics and Management, Ural Federal University, Yekaterinburg 620002, Russia; 3 Laboratory of Complex Electrophysic Investigations, Institute of Electrophysics, Ural Branch of the Russian Academy of Sciences, Yekaterinburg 620016, Russia; 4 Department of Physical and Inorganic Chemistry, Institute of Natural Sciences and Mathematics, Ural Federal University, Yekaterinburg 620002, Russia

**Keywords:** solid oxide fuel cells, electrophoretic deposition, MIEC electrolyte, barrier layer, co-doped CeO_2_, doped BaCeO_3_

## Abstract

The strategy to increase the performance of the single solid oxide fuel cell (SOFC) with a supporting membrane of Ce_0.8_Sm_0.2_O_1.9_ (SDC) electrolyte has been implemented in this study by introducing a thin anode barrier layer of the BaCe_0.8_Sm_0.2_O_3_ + 1 wt% CuO (BCS-CuO) electrolyte and, additionally, a modifying layer of a Ce_0.8_Sm_0.1_Pr_0.1_O_1.9_ (PSDC) electrolyte. The method of electrophoretic deposition (EPD) is used to form thin electrolyte layers on a dense supporting membrane. The electrical conductivity of the SDC substrate surface is achieved by the synthesis of a conductive polypyrrole sublayer. The kinetic parameters of the EPD process from the PSDC suspension are studied. The volt-ampere characteristics and power output of the obtained SOFC cells with the PSDC modifying layer on the cathode side and the BCS-CuO blocking layer on the anode side (BCS-CuO/SDC/PSDC) and with a BCS-CuO blocking layer on the anode side (BCS-CuO/SDC) and oxide electrodes have been studied. The effect of increasing the power output of the cell with the BCS-CuO/SDC/PSDC electrolyte membrane due to a decrease in the ohmic and polarization resistances of the cell is demonstrated. The approaches developed in this work can be applied to the development of SOFCs with both supporting and thin-film MIEC electrolyte membranes.

## 1. Introduction

The design of solid oxide fuel cells (SOFCs) with a supporting electrolyte membrane is considered to be promising in terms of advantages, such as mechanical strength of the structure, reliable separation of gas channels, and the possibility of reducing the polarization resistance due to porous, thin-film electrodes [1,2,3,4]. Despite a large number of works on thin-film electrolytes obtained by different methods [5,6,7], the development of SOFCs with a carrier electrolyte highlights directions for the creation of efficient and time-reliable SOFCs. The main drawback of the carrier electrolyte SOFC design is the significant contribution of the ohmic resistance of the electrolyte due to its thickness; therefore, many efforts have been made to search for efficient electrolyte materials for operation in the intermediate and low-temperature range [8,9].

Ceria based solid electrolytes show promising conductivity in the temperature range of 500–700 °C. In addition, the formation of nanocomposites based on doped/co-doped ceria allows further reduction in the working temperature [10,11,12]. The main disadvantage of CeO_2_ electrolytes is their reduction in the hydrogen atmosphere of the anode channel with the appearance of n-type electronic conductivity [10,13]. The electronic leakage current significantly reduces the open circuit voltage (OCV) of the SOFC cell, which worsens its energy efficiency and fuel utilization ratio [14].

Among the known methods to eliminate the internal leakage current in SOFCs with MIEC electrolyte membranes is the use of anode barrier layers based on materials with pure ionic conductivity in reducing atmospheres, for example, stabilized ZrO_2_ [15,16] or doped BaCeO_3_ [17,18,19]. Solid electrolytes based on ZrO_2_ have the advantage of chemical and phase stability independent of oxygen partial pressure and the presence of hazardous gases [20]. However, the manufacture of ZrO_2_/CeO_2_ electrolyte membranes by the methods involving high-temperature sintering is complicated by the formation of secondary phases and the occurrence of delamination at the electrolyte/barrier layer interface due to a mismatch in thermo-mechanical properties [21,22,23]. The use of barrier layers based on doped barium cerate has advantages due to the thermomechanical properties of these materials close to those of doped CeO_2_ and the higher conductivity and lower activation energy in the IT range compared to the ZrO_2_-based electrolytes [19,24,25].

Considering Sm-doped BaCeO_3_ (BCS) as a potential anode barrier layer compatible with Sm-doped CeO_2_ (SDC), the most conductive of the doped ceria electrolytes [26], a decrease in electrolyte membrane conductivity should be considered due to the lower ionic conductivity of BCS compared to SDC [27]. Thus, despite the increased OCV, the power output of the cell with a double-layer electrolyte membrane may be lower than that of a cell with a single-layer SDC membrane [28].

An important factor in achieving higher SOFC power output is the balance between the OCV and the total (ohmic and polarization resistance) of the cell. In particular, the high polarization resistance of Pt electrodes in contact with BaCeO_3_-based materials can significantly contribute to the degradation of cell performance [28,29]. Therefore, the development of electrodes that are highly active in the IT range and compatible with CeO_2_ and BaCeO_3_ electrolytes is of great importance [30,31,32]. The rate of oxygen reduction reaction (ORR) at the cathode can be increased using cathode sublayers possessing higher ionic conductivity compared to the parent electrolyte, for example, doped Bi_2_O_3_ electrolytes [33,34] or those with mixed oxygen ion and *p*-type electron conductivity [35,36,37,38]. For example, mixed valence Pr^3+/4+^ used as a co-dopant was found to induce partial electronic conductivity in Gd-doped CeO_2_ in an air atmosphere, increasing the grain boundary conductivity by about two orders compared to the base solid solution [39,40]. The presence of a Pr-enriched phase at the electrolyte grain boundaries was shown to have a positive effect on the electrochemical activity of Pt electrodes in contact with Ce_0.80_Gd_0.18_Pr_0.02_O_2−δ_ electrolyte [41]. The performance of the SOFC with an Sm-doped ceria electrolyte was improved by co-doping with Pr, mainly due to a decreased polarization resistance of the electrolyte–electrode interface [35]. However, the authors observed a decrease in the OCV value for the cell with the Pr co-doped electrolyte.

The method of electrophoretic deposition (EPD) allows the formation of multilayer electrolyte membranes with precise control of the thickness of the deposited layers. EPD is suitable for the formation of coatings of complex compositions on substrates of arbitrary shape [42,43,44]. The peculiarity of the EPD process is the formation of coating in the liquid suspension under the influence of an external electric field. In contrast to sputtering and pulsed laser deposition (PLD) methods, the EPD method does not require expensive technological equipment and has higher deposition rates (up to 3 microns per minute). However, coatings obtained by the EPD method require high-temperature sintering. The sintering procedure is accompanied by shrinkage of the deposited powder material and substrate material and by diffusion processes of element redistribution, in contrast to sputtering and PLD methods, which do not require high sintering temperatures. To perform the EPD process on a dense, non-conducting substrate, it is necessary to make its surface electrically conductive. Known options for creating conductive sublayers on non-conductive substrates include the deposition of carbon [45] or platinum [46] and the synthesis of conductive polymer polypyrrole (PPy) on the substrate surface [19,47,48]. 

The aim of the present work is to investigate the possibility of increasing the performance of a single SOFC with a supporting Ce_0.8_Sm_0.2_O_1.9_ (SDC) electrolyte membrane by introducing a barrier layer of a BaCe_0.8_Sm_0.2_O_3_ + 1 wt% CuO (BCS-CuO) electrolyte on the anode side and a modifying layer of a Ce_0.8_Sm_0.1_Pr_0.1_O_1.9_ (PSDC) electrolyte on the cathode side. Electrophoretic deposition was used to form the thin electrolyte layers on a dense supporting membrane. In order to implement the EPD process, a conductive polymer polypyrrole sublayer was synthesized to provide electrical conductivity of the SDC substrate surface under the EPD conditions. The features of a PSDC suspension preparation, its characteristics, and the kinetic parameters of the EPD process from the prepared suspension were studied for the first time. A comparative study of the obtained SOFC cells with two different electrolyte membranes was performed: SDC membrane with a BCS-CuO blocking layer on the anode side (BCS-CuO/SDC) and that with the BCS-CuO blocking layer on the anode side and the PSDC modifying layer on the cathode side (BCS-CuO/SDC/PSDC). A peculiarity of this work was the use of a LSFC-SDC/LNF-EDB-CuO ceramic cathode and a NiO-BCGCu cermet anode, possessing excellent electrochemical activity in the IT-range, which have been developed in our recent studies. The effect of increasing the power output of the cell with the BCS-CuO/SDC/PSDC electrolyte membrane due to a decrease in the ohmic and polarization resistances of the cell was demonstrated. The approaches developed in this work can be applied to the subsequent development of SOFCs with a supporting MIEC electrolyte membrane. 

## 2. Materials and Methods

### 2.1. Synthesis and Characterization of the Electrolytes 

Ce_0.8_Sm_0.2_O_1.9_ (SDC) electrolyte was synthesized by a solid-state reaction (SSR) method using CeO_2_ (99.9 %wt) and Sm_2_O_3_ (99.9 %wt) (Reakhim, Moscow, Russia) as the starting reagents. After mixing in a PM 100 planetary mill (Retsch, St. Petersburg, Russia) and drying, the reagents were calcined at 950 °C (10 h) and 1050 °C (10 h) with the intermediate grinding in the agate mortar. The as-synthesized powder was ball-milled for 1 h, dried and compacted at 300 MPa into the disk-shaped samples and sintered at 1600 °C (3 h). The relative density of the SDC electrolyte membranes, defined from their size and weight, reached approximately 97% of the theoretical value calculated for the XRD data. 

The choice of the anode barrier layer composition was based on the compatibility criteria. It is known that Gd or Nd-doping allows obtaining BaCeO_3_-based materials with the highest conductivity level [25]. However, in our preliminary studies, we did not manage to successfully sinter the electrophoretically deposited Gd-doped BaCeO_3_ films on the SDC substrate. Therefore, Sm-doped BaCeO_3_ was chosen for the barrier layer formation, which characterizes excellent chemical compatibility with SDC, demonstrated in a number of studies [49,50]. CuO additive was used to increase the film sinterability without deteriorating its conductivity [28]. The BaCe_0.8_Sm_0.2_O_3_ + 1 wt% CuO (BCS-CuO) electrolyte for the anode barrier layers was synthesized by a citrate–nitrate combustion (CNC) route using BaCO_3_ (99.0 %wt), Ce(NO_3_)_3_∙6H_2_O (99.9 %wt), Sm(NO_3_)_3_·6H_2_O (99.9 %wt), and CuO (99.0 %wt) (Reakhim, Moscow, Russia) as the starting reagents. CuO and BaCO_3_ were dissolved in a minimum amount of diluted HNO_3_, then mixed with the metal nitrates dissolved in distilled water. Citric acid (chelating agent) and glycerin (organic fuel) were introduced into the mixture in a ratio of 0.5:1.5 per 1 mole of the mixed oxide. The mixture was heated up to 100 °C under constant stirring, then 10 % ammonia solution was added to establish pH value equal to 7. In the next synthesis step, the solution was evaporated until a xerogel formation and following self-ignition. The obtained powder was calcined at 700 °C (1 h) to eliminate organic residues. To complete the crystal structure formation, the precursor was calcined at 1050 °C (5 h) and 1150 °C (5 h) with the intermediate grinding in the agate mortar. The BCS-CuO powder used for the suspension preparation was milled for 3 h in the planetary mill.

Ce_0.8_Sm_0.1_Pr_0.1_O_1.9_ (PSDC) electrolyte for the cathode sublayers was synthesized by a citrate-nitrate combustion route using Ce(NO_3_)_3_∙6H_2_O (99.9 %wt), Sm(NO_3_)_3_·6H_2_O (99.9%wt), and Pr(NO_3_)_3_·6H_2_O (99.9 %wt) (Reakhim, Moscow, Russia) as the starting reagents. The reagents were dissolved in distilled water. Citric acid and glycine were introduced into the mixture in a ratio of 0.6:0.8 per 1 mole of the mixed oxide. The mixture was heated until a gel formation and following self-ignition. The obtained powder was calcined at 800 °C (5 h) to eliminate organic residues and complete the crystal structure formation. 

The XRD analysis of the obtained powders was completed using an XRD-7000 diffractometer (Shimadzu, Kyoto, Japan) in a CuKα radiation, in an 2θ angle range of 25–80° with a step of 0.02° and a fixed time of 5 sec at each point. Phase identifications were performed using PDF-4 database (ICDD, Newtown, CT, USA, Release 2018). The parameters of the crystal structure of the obtained materials were refined using FullProf Suite software [51]. The specific surface area of the powders was determined using a SORBI N 4.1 Instrument (Meta, Novosibirsk, Russia). The morphology of the BCS-CuO and PSDC powders was investigated using a JSM-6390 LA scanning electron microscope (JEOL, Tokyo, Japan).

### 2.2. Synthesis of the Electrode Powders

The main criteria for the electrode choice were their excellent electrochemical activity at decreased operating temperatures. La_0.6_Sr_0.4_Fe_0.8_Co_0.2_O_3−δ_ (LSFC) is the most often used in SOFCs with doped CeO_2_ electrolyte membranes due to its high conductivity and excellent electro-catalytic activity to ORR in the IT-range [52,53]. It is generally accepted that the electrochemical performance of LSFC can be further improved by mixing the cathode material with an electrolyte [35,54,55]. Recently, Pikalova et al. reported on the possibility of the additional activation of the composite electrodes using CuO and Bi_2_O_3_-Er(Y)_2_O_3_ additives [32,56]. Therefore, for this study, in contact with the SDC and PSDC electrolytes, a bilayer cathode with the 60 wt% La_0.6_Sr_0.4_Fe_0.8_Co_0.2_O_3−δ_ (LSFC)-40 wt% SDC functional layer and 87 wt% LaNi_0.6_Fe_0.4_O_3−δ_ (LNF)-10 wt% Bi_1.6_Er_0.4_O_3_ (EDB)-3 wt% CuO collector was chosen. 

The LSFC powder was synthesized by a citrate-nitrate combustion route using La_2_O_3_ (99.99 %wt), SrCO_3_ (99.0 %wt) (Reakhim, Moscow, Russia), Fe(NO_3_)_3_·9H_2_O (>98 %wt), and Co(NO_3_)_2_·6H_2_O (>99 %wt) (LenReactiv, St. Petersburg, Russia) as the starting reagents and citric acid as a chelating agent and an organic fuel taken in a ratio of 1:1 to the mixed oxide. The powder obtained after combustion was calcined at temperatures of 950 (10 h) and 1100 °C (10 h) with the intermediate grinding in the agate mortar to remove organic residues and form a perovskite crystal structure. 

The LNF electrode material was synthesized by a modified Pechini (MP) method using La(NO_3_)_3_·6H_2_O (99.9 %wt) (Reakhim, Moscow, Russia), Ni(NO_3_)_2_·6H_2_O (99.4 %wt), and Fe(NO_3_)_3_·9H_2_O (>98 %wt) (LenReactiv, St. Petersburg, Russia) as the starting reagents. The nitrates were dissolved in distilled water, then citric acid and ethylene glycol were added as a chelating agent and an organic fuel, respectively. The solution was evaporated and thermally decomposed at 450 °C. The obtained precursor was ball-milled and calcined at 900 °C (5 h). The CuO nanopowder used as an additive to the collector layer was obtained by an electric explosion of a copper wire in O_2_ [57].

The SDC solid electrolyte for the composite electrodes was obtained using a synthesis procedure similar to that for the PSDC electrolyte (Section 2.1). The synthesis of EDB electrolyte was carried out by nitrate combustion using Er_2_O_3_ (99.9 %wt) and Bi(NO_3_)_3_·5H_2_O (99 %wt) (Reakhim, Moscow, Russia) as the starting reagents with citric acid as an organic fuel taken in a ratio of 1:1 to the mixed oxide. The precursor powder was calcined at 600 °C (5 h).

According to the recent studies presented by Antonova et al. group [31], the anode comprising 56 wt% NiO and 44 wt% BaCe_0.89_Gd_0.1_Cu_0.01_O_3−δ_ (BCGCu) exhibited the polarization resistance as low as 0.7 Ω cm^2^ at 600 °C in wet hydrogen in contact with a BaCeO_3_-based protonic conductor. This perspective composition was taken as an anode in the present study. BCGCu was synthesized from BaCO_3_ (99.0 %wt), CeO_2_ (99.99 %wt), Gd_2_O_3_ (99.99 %wt) and CuO (99.0 %wt) (Reakhim, Moscow, Russia) as the starting reagents. Mixing the oxides and mechanical activation between the synthesis stages was performed in the planetary mill for 1 h. The synthesis was performed at 1100 (2 h) and 1450 (5 h, in tablets). After the synthesis the samples were crushed, sieved (≤1 μm) and ball-milled for 1 h. The BCGCu and NiO (99 wt%) (Ormet, Yekaterinburg, Russia) were mixed in the planetary mill for 1 h.

### 2.3. Preparation and Characterization of PSDC and BCS-CuO Suspensions for EPD

A mixed dispersion medium comprising isopropanol and acetylacetone taken in a 70:30 vol. ratio was used for the suspensions’ preparation. The suspensions of the BCS-CuO and PSDC powders with a concentration of 10 g/L were prepared by ultrasonic treatment (UST) in an UZV-13/150-TH ultrasonic bath (Realtek, Yekaterinburg, Russia) at the power of 210 W and the operating frequency of 22 kHz for 5–125 min. The constant temperature in the bath of 25 °C was maintained by water exchange. To ensure deposition, molecular iodine was added to the BCS-CuO suspension in an amount of 0.4 g/L. Electrokinetic zeta potential and pH of the suspensions were measured using a DT-300 analyzer (Dispersion Technology, Bedford Hills, NY, USA).

### 2.4. Electrophoretic Deposition and Characterization of the PSDC and BCSCuO Films 

Before the deposition, the SDC electrolyte membranes were polished to the thickness of 550 μm using a diamond polishing disk (Yuguan Abrasive Co., Dacheng, Hebei Province, China) with following cleaning in the ultrasonic bath for 10 min and firing at 900 °C for 1 h. The EPD modes during deposition were controlled using a specialized laboratory setup (IEP UB RAS, Yekaterinburg, Russia). A conductive polymer film of polypyrrole was synthesized on the SDC electrolyte surface by chemical polymerization of pyrrole in an aqueous solution of ammonium persulfate served as an oxidizing agent (98%, 0.03 M); and sodium salt of p-toluenesulfonic acid, served as a dopant (97.5%, 0.03 M), and a pyrrole monomer (98%, 0.03 M), as described elsewhere [19]. The EPD of the PSDC and BCS-CuO thin films was carried out at the constant voltage. A disk-shaped SDC electrolyte membrane with the surface area of approximately 1.2 cm^2^ was placed on the cathode electrode in the EPD cell. A stainless-steel disk with an area of 1 cm^2^ was used as a counter electrode; the distance between the electrodes was 1 cm. The current strength during the deposition was measured using an Intelligent Digital Multimeter UNI-T UT71E (Uni-Trend Technology, Shanghai, China). The layers deposited on the SDC substrate were sintered in air at different temperatures using a high-temperature furnace LHT-04/18 (Nabertherm, Lilienthal, Germany).

The SEM examination of the deposited films was carried out using a JSM-6390 LA scanning electron microscope (JEOL, Tokyo, Japan) equipped with an energy dispersive X-ray microanalysis (EDX) system at a high voltage of 10 kV. The morphology of the green deposited films was inspected using an VS-520 optical microscope (STAT, Yekaterinburg, Russia). The thickness of green deposited films was estimated from the deposition weight, the film surface area and the theoretical density of PSDC and BCS-CuO calculated from the XRD data. The actual thickness of the sintered films was evaluated from the SEM cross-sectional images.

### 2.5. Single-Cell Fabrication and Electrochemical Characterization

To fabricate the cells for the electrochemical study, cathodes and anodes were consequently deposited on the SDC electrolyte membranes with the sintered barrier coatings. Electrode slurries were prepared on the base of the electrode powders (Section 2.2) with the addition of ethanol and polyvinyl butyral binder. The NiO-BCGCu anode was deposited with the thickness of 40 μm and sintered at 1300 °C (2 h). The LSFC-SDC functional cathode layer was deposited with a thickness of 30 μm and sintered at 1000 °C (2 h). As a second layer, the LNF-EDB-CuO collector was deposited with the thickness of 40 μm and sintered at 900 °C (2 h). The electrodes were activated by infiltration with an aqueous solution of Ce(NO_3_)_3_∙6H_2_O on the anode side and an ethanol-based Pr(NO_3_)_3_∙6H_2_O solution on the cathode side, followed by calcination at 600 °C (1 h). 

The measurement cell was made of YSZ tube with deposited Pt electrodes, which served as electrochemical pump and oxygen partial pressure sensor. The sample for measurement was installed on the top of the YSZ tube (with the cathode side facing inside the tube) and fixed using a high-temperature sealant by heating up to 930 °C (10 min). When the sensing cell was heated in the wet air flow (3% H_2_O, 5 L/h flow rate) at 600 °C, the oxidizing atmosphere in the anode channel (outside the tube) was gradually replaced by Ar (5 L/h), then by humidified hydrogen (5% H_2_O, 5 L/h). After the complete reduction in the NiO-BCGCu anode, the EMF values measured by the sensor on the YSZ tube reached approximately 1120 mV (at 600 °C), indicating good settling strength of the sample fixed on the measuring cell. 

The electrochemical study was performed in the temperature range of 650 °C to 800 °C by measuring OCV and impedance spectra under OCV conditions in the frequency range of 10^6^–0.1 Hz at an applied alternating signal of 30 mV and volt-ampere dependencies and power output using a B2901A current source/measuring unit (Keysight, Colorado Springs, CO, USA) and a Parstat 3000A potentiostat/galvanostat (Ametek Scientific Instruments, Oak Ridge, TN, USA). 

## 3. Results and Discussion

### 3.1. Characterization of the Initial Electrolyte and Electrolyte Powder Materials and Suspension Preparation for the EPD Process

The results of the XRD characterization of the materials synthesized for the supporting electrolyte membranes, electrolyte barrier layers, and electrode preparation are represented in Table 1. The XRD data with the refinement are given in Figure S1. 

**Figure 1:**
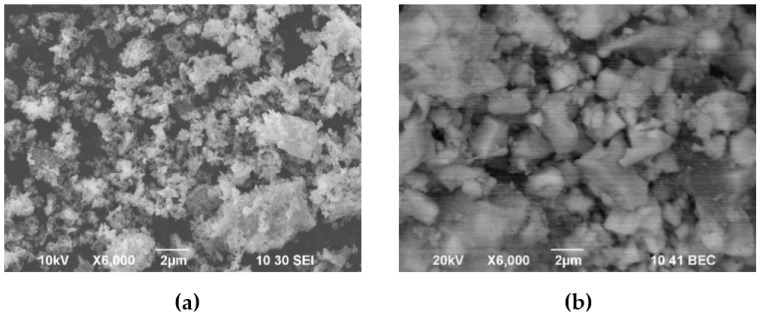
Morphology of the initial electrolyte powders used for the suspension preparation: PSDC (T_calc_ = 800 °C) (SEM image) (**a**); BCS-CuO (T_calc_ = 1150 °C) (SEM image) (**b**).

SEM images of the BCS-CuO and PSDC powders used for the suspension preparation are shown in Figure 1. The PSDC powder was represented by the presence of loose aggregates up to 1–2 µm and fine particles less than 1 µm in size (Figure 1a). The BCS-CuO powder morphology (Figure 1b) was characterized by large dense particles up to 3 µm in size. Despite ball-milling, this material had a poorly developed surface due to the higher temperature required to achieve a single-phase state. As the specific surface area values (Table 1) for PSDC and BCS-CuO differed significantly, different approaches were required to prepare stable suspensions based on these powders.

The process of electrophoresis in suspension is characterized by electrokinetic or zeta potential, which determines the amount of excess charge on particles in suspension due to the formation of an electric double layer during specific adsorption of potential-determining ions (protons) on the surface of particles dispersed in the medium. The zeta potential value determines the stability of the suspension and its applicability for deposition. In turn, the pH value in the suspension characterizes the concentration of free protons and, therefore, affects the conductivity of the suspension [58,59]. Zeta potential and pH were measured in the basic suspensions of PSDC and BCS-CuO and in the BCS-CuO suspension with the addition of iodine at a concentration of 0.4 g/L, ultrasonically treated for 5 and 125 min (Table 2). For the PSDC suspension, prolonged sonication for 125 min resulted in an increase in the zeta potential value from +16 mV to +23 mV with a concomitant increase of pH from 5.5 to 6.4, which may be due to the loose nature of the fine PSDC powder used. The BCS-CuO suspension was characterized by a lower initial zeta potential value of +11 mV. 

According to the zeta potential value, the PSDC suspension can be classified as a stable suspension, while the BCS-CuO suspension belongs to relatively stable suspensions, according to the criteria proposed in [59]. The positive sign of the zeta potential corresponds to a positive charge on the particles; thus, the cathodic deposition process is realized. It is worth noting that the UST duration did not affect the zeta potential value, although the pH decreased from 5.1 to 4.2. 

The preliminary EPD experiments on a Ni-foil showed that the PSDC suspension was ready for use after 125 min of UST, while no deposition occurred from the basic BCS-CuO suspension. To initiate EPD from the BCS-CuO suspension, the introduction of molecular iodine was required. The addition of iodine to the BCS-CuO suspension did not change the zeta potential but decreased the pH value from 4.2 to 3.7. Molecular iodine has been used for the suspension modification in a number of studies [60,61,62]. The addition of iodine leads to an increase in the concentration of free protons in the suspension, which increases its conductivity and facilitates the deposition process. Therefore, the modified BCS-CuO suspension was used for further experiments.

### 3.2. EPD from the PSDC and BCS-CuO Suspensions on a Ni-Foil Model Electrode: Selection of Deposition Modes

**Figure 2:**
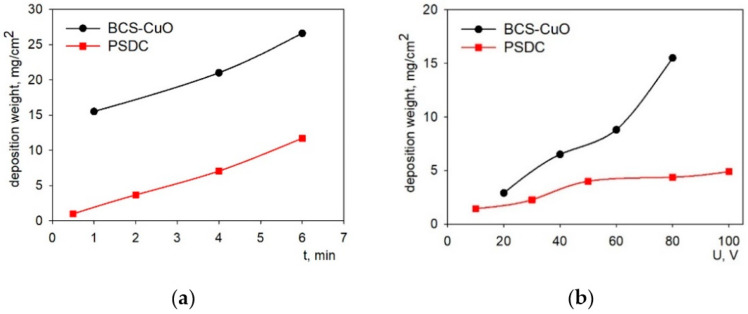
Dependences of the deposition weight on time at the constant voltage for the BCS-CuO suspension (80 V) and the PSDC suspension (40 V) (**a**); dependence of the deposited mass on the voltage at a constant deposition time of 1 min (**b**).

The deposition weight versus time dependencies were obtained at a constant voltage of 80 V for the modified BCS-CuO suspension and at 40 V for the PSDC suspension (Figure 2a). The choice of deposition voltages for the BCS-CuO and PSDC suspensions was based on the condition for obtaining a uniform continuous coating. Namely, the best results in terms of uniformity of the BCS-CuO coating corresponded to a voltage of 80 V, while to obtain a uniform PSDC coating, it was necessary to perform the EPD process at a voltage of 40 V. The obtained time dependences for both suspensions were characterized by an almost linear increase in weight. At the same time, the growth of the deposition weight as a function of the applied voltage at a constant time equal to 1 min was non-linear (Figure 2b). Deposition from the BCS-CuO suspension was more intense compared to the PSDC suspension due to both the introduction of molecular iodine into the BCS-CuO suspension and a higher deposition voltage. To obtain continuous BCS-CuO coatings with a deposition weight of ~15 mg/cm^2^, corresponding to a thickness of ~20 µm (calculated considering the theoretical BCS-CuO density of 6.33 g cm^−3^), the deposition time at 80 V was 1 min. To obtain continuous PSDC coatings with a thickness of 5 µm, corresponding to a deposition weight of ~4 mg cm^−2^ and the theoretical density of 7.12 g cm^−3^, the deposition time at 40 V was about 2 min. The required thicknesses of the anode and cathode barrier layers were chosen based on the results of our previous study [19]. 

**Figure 3:**
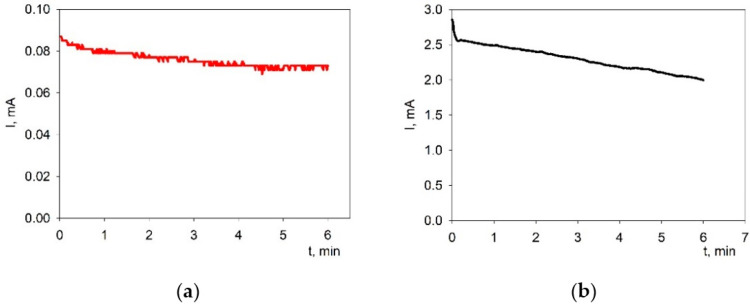
Dependence of the current strength on the deposition time during EPD from suspensions: PSDC (at 40 V) (**a**); BCS-CuO (at 80 V) (**b**).

The obtained dependences of the current strength on the deposition time during EPD from the suspensions of PSDC (voltage 40 V) and BCS-CuO (voltage 80 V) showed a slight decrease in the current strength with time (Figure 3). The magnitude of the current strength in the BCS-CuO suspension was significantly higher than that in the PSDC suspension due to its higher conductivity due to the addition of iodine, which is consistent with more intense deposition from the BCS-CuO suspension.

### 3.3. EPD of BCS-CuO Anode Barrier and PSDC Cathode Layers on Dense SDC Membranes with Predeposited PPy Sublayers

**Figure 4:**
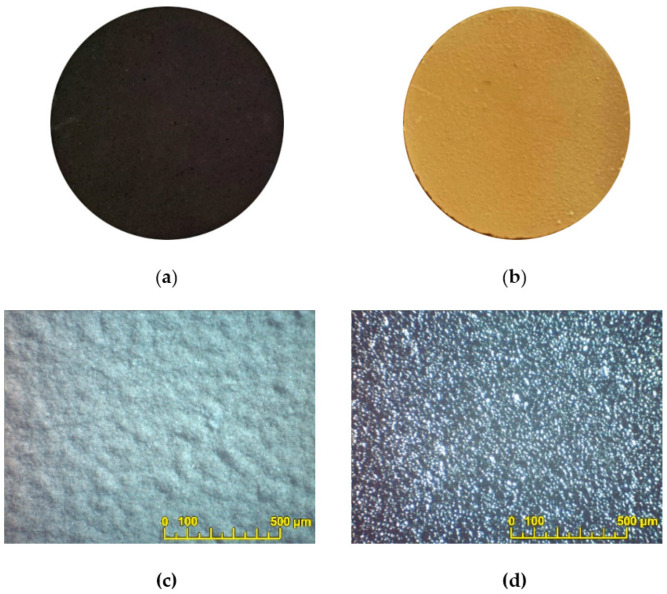
Illustration of the process of the BCS-CuO coating formation on the dense SDC membrane: the SDC membrane with PPy coating synthesized on its surface (anode side) (**a**); photograph of the sample with a deposited green BCS-CuO coating (**b**); optical image of the BCS-CuO coating before sintering (**c**); optical image of the BCS-CuO coating after sintering at 1530 °C for 5 h (**d**).

The EPD modes from the PSDC and BCS-CuO suspensions selected on the base of the deposition experiments on the Ni-foil were further adapted for EPD on the supporting SDC electrolyte membranes with pre-deposited PPy sublayers. First, the EPD method was used to deposit a BCS-CuO layer on the SDC electrolyte membrane with a PPy sublayer synthesized on the anode side (Figure 4a). EPD was carried out in a constant voltage mode of 80 V for 6 min. The BSC-CuO coating was dried in a Petri dish at room temperature for 24 h. No visible defects were observed in the BCS-CuO coating after drying (Figure 4b,c). The thickness of the dried coating was 18 µm. When performing the EPD of the BCS-CuO film on the SDC substrate with the PPy sublayer, it was necessary to increase the deposition time compared to the deposition on the Ni foil due to the lower conductivity of the PPy sublayer (500 S m^−1^) compared to the Ni foil (11.5 × 10^6^ S m^−1^). The BCS-CuO coating was sintered at a temperature of 1530 °C for 5 h. According to the optical microscopy study, the dense, crack, and pore-free BCS-CuO coating was obtained (Figure 4d). 

**Figure 5:**
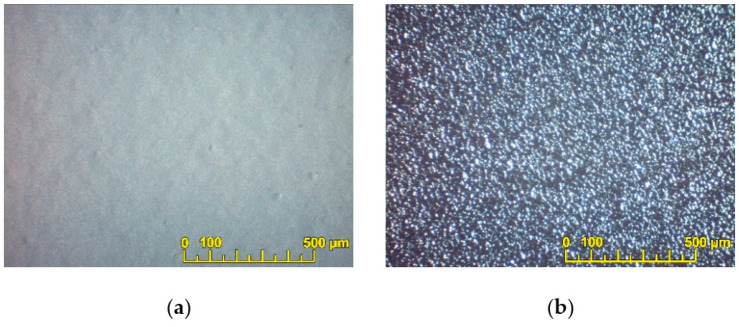
Optical images of the PSDC coating formed on the cathode side of the SDC membrane: the green PSDC coating after drying (**a**); the PSDC coating sintered at 1450 °C for 5 h (**b**).

The second sample with an 18 μm thick BCS-CuO layer on the anode side of the SDC electrolyte membrane was fabricated using a similar scheme. After the sintering of the BCS-CuO barrier layer, PPy synthesis was performed on the cathode side of the SDC membrane. EPD of the PSDC coating was carried out in a constant voltage mode of 40 V for 3 min. The coating was dried in a Petri dish for 24 h. The optical image of the green PSDC coating after its drying is shown in Figure 5a. The thickness of the dried coating was 6 µm. After the sintering at 1450 °C for 5 h, the dense, defect-free PSDC coating was obtained (Figure 5b). 

### 3.4. Comparative Electrochemical Testing the Single SOFCs with the Supporting SDC Electrolyte Membrane and Electrolyte Coatings Applied by EPD on the Anode and on the Anode/Cathode Side

**Figure 6:**
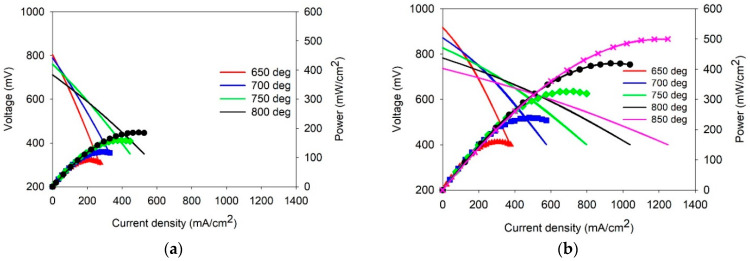
Volt-ampere characteristics and power output of the single SDC-supported cells (550 μm): with the BCS-CuO anode barrier layer (18 μm) (**a**); with the BCS-CuO anode barrier layer (18 μm) and the PSDC cathode layer (6 μm) (**b**).

According to the procedure of the electrode deposition (Section 2.5), two single cells were fabricated: NiO-BCGCu (40 µm)/BCS-CuO (18 µm)/SDC (550 µm)/LSFC-SDC (30 µm)/LNF-EDB-CuO (40 µm) (denoted as SDC1); NiO-BCGCu (40 µm)/BCS-CuO (18 µm)/SDC (550 µm)/PSDC (6 µm)/LSFC-SDC (30 µm)/LNF-EDB-CuO (40 µm) (denoted as SDC2). Volt-ampere characteristics and power output for the cells in the range of 650–800 °C are shown in Figure 6. 

The SDC1 cell showed specific power densities (SPD) of 90–186 mWcm^−2^ at the temperature of 650–800 °C. It is interesting to note that despite the thickness of the electrolyte equal to 550 μm, the power density values obtained on the SDC1 were similar to those obtained for the single SOFC with the thin-film SDC electrolyte (18 μm) and the barrier BCS-CuO layer of 13 μm formed by EPD on the Ni-BCS-CuO supporting anode (800 μm) equal to 50–160 mW cm^−2^ at 650–750 °C, respectively [28]. Obviously, this again confirms that the polarization resistance of the supporting anode can be a critical factor in the deterioration of cell performance. Thus, in some cases, the use of thin electrodes with high electrochemical activity may be advantageous for an electrolyte-supported cell over a cell with a thin-film electrolyte membrane on a thick supporting electrode [13,63]. However, it should be noted that the OCV values for the SDC1 cell, which ranged 0.81–0.71 V at 650–800 °C, were close to those typical for the SDC electrolyte without barrier layers [64,65], while in the case of the thin-film SDC, the application of the BCS-CuO barrier layer resulted in the significant increase in the OCV level up to 1.05–0.95 V at temperatures of 600–700 °C [28]. A similar effect of increasing OCV was observed when SDC thin-film electrolyte membranes were deposited on the supporting Ni-cermet anode with a proton-conducting electrolyte in its content (1.047–1.004 V at 600–700 °C for SDC (30 μm) on Ni-BaZr_0.1_Ce_0.7_Y_0.1_Yb_0.1_O_3−δ_ [66]; 1.06–0.92 V at 650–750 °C for SDC (30 μm) modified with Co, Ti, and Al sintering additives on the supporting Ni-BCS anode [67]). The specific power of the SDC2 cell with both the BCS-CuO anode barrier layer and the PSDC cathode layer reached 159–419 mW cm^−2^ at temperatures of 650–800 °C, respectively, and was significantly higher than that of the SDC1 cell. It also exceeded the performance of the cell with a supporting SDC electrolyte of the similar thickness (500 μm) with a BaCe_1−x_Sm_x_O_3−δ_ anode layer and with a Sm_0.5_Sr_0.5_CoO_3_ cathode (76–278 mW cm^−2^ at 600–800 °C) [18]. In contrast to the tendency to reduce the OCV level when using the Pr-co-doped SDC electrolyte membrane down to 0.783–0.717 V compared to that for the SDC membrane ranged 0.838–0.714 V (650–750 °C) [35], in the case of thin-film PSDC on the cathode side (SDC2 cell) the OCV values were 0.918, 0.872, 0.827, and 0.782 V at temperatures of 650, 700, 750, and 800 °C, respectively, and were higher than those of the SDC1 cell. Table 3 summarizes the results obtained in this study in comparison with the literature data. The electrolyte membranes obtained by EPD have been marked.

**Figure 7:**
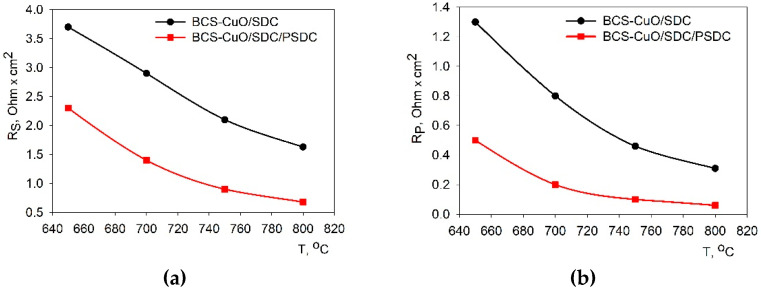
Results of the impedance spectroscopy study under the OCV conditions of the cells with different electrolyte membranes: temperature dependence of the ohmic resistance—(**a**); temperature dependence of the total polarization resistance—(**b**).

The analysis of the spectra obtained under the open circuit conditions made it possible to determine the *R_hf_* value (series resistance) as an inflection point in the impedance spectrum on the Nyquist diagram between its high-frequency part (electrolyte contribution) and mid-low frequency part (electrode contribution). The *R_η_* values, corresponding to the total polarization contribution of the electrodes, were derived from the measurements of the total cell resistance under *dc* current, *R_dc_*, as *R_η_ = R_dc_* − *R_hf_*. Using these values and taking into account that the thick electrolyte membrane is the major contributor to the series resistance, the ohmic resistance and the total polarization resistance of the electrodes were calculated as *R_s_ = S × R_hf_* and *R_p_ = S × R_η_*, where *S* is the total effective area of the electrodes on both sides of the cell. Nyquist diagram of the impedance data obtained under open circuit values is shown in Supplementary materials, Figure S2. The serial resistance values were subtracted from the EIS data for better comparison of the spectra obtained over the wide temperature range. The *R_s_* and *R_p_* values at different temperatures are summarized in Figure 7a,b.

From the obtained results, it can be concluded that the PSDC layer on the cathode side greatly contributed to a decrease in the ohmic resistance of the electrolyte membrane measured in the SOFC mode (Figure 7a), related to an increase in the ionic conductivity of the PSDC electrolyte, which was documented in a number of studies [35,69,70]. Particularly, an increase in the number of oxygen vacancies in the Pr co-doped SDC was demonstrated by the Raman spectroscopy method [35,70].

**Figure 8:**
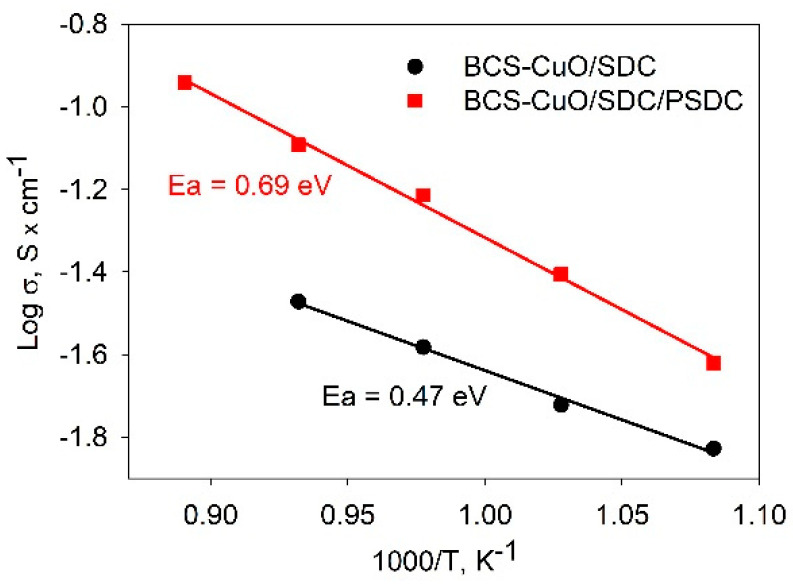
Arhenius dependences of the conductivity of BCS-CuO/SDC/PSDC and BCS-CuO/SDC electrolyte membranes calculated from the EIS data obtained in OCV conditions for the SDC1 and SDC2 cells.

The PSDC layer had also a significant depolarizing effect on the polarization resistance of the cathode, such that the total *R_p_* value decreased to 0.5, 0.2, 0.1, and 0.05 Ohm cm^2^ at temperatures of 650, 700, 750, and 800 °C, respectively (Figure 7b). The temperature dependences of the conductivity of the electrolyte membranes of the SDC1 and SDC2 samples in Arrhenius coordinates are shown in Figure 8. The SDC1 sample was characterized by low conductivity values and, at the same time, a low activation energy equal to 0.47 eV, which is typical for Sm-doped BaCeO_3_ electrolytes [28,50,71]. Thus, in the absence of a modifying cathode layer, it was the electrical properties of the BaCeO_3_ film that determined the character of the conductivity of the SDC-supporting electrolyte membrane. The activation energy of the conductivity of the SDC2 sample (Ea = 0.69 eV) was close to the activation energy characteristic of SDC [10,11,69]. 

After several days of testing in the SOFC mode, the SDC2 cell was cracked to perform microstructural studies. The results are shown in Figure S3. The SDC-based electrolyte membrane with the PSDC cathode and BCS-CuO anode barrier layers has a well sintered dense structure. The PSDC and BCS-CuO layers are fused with the SDC substrate; the boundaries between the SDC substrate and the layers are indistinguishable. This is due to the composition of the layers close to the main membrane composition. The bilayer cathode and anode retained their integrity and good adhesion to the electrolyte membrane and between the constituent layers. The anode structure appears rather dense, which can be improved by lowering the sintering temperature. Nevertheless, the use of NiO-BCGCu cermet anode and LSFC-SDC/LNF-EDB-CuO multilayer ceramic cathode showed their superior efficiency compared to the Pt-activated electrodes [19,28].

## 4. Conclusions

The strategy to enhance the performance of the single SOFC with the supporting SDC electrolyte membrane was implemented in this study. The thin barrier layer based on of the BCS-CuO electrolyte was deposited on the anode side of the SDC membrane (550 μm) as a barrier layer to protect the ceria-based electrolyte from reduction under reducing conditions. In addition, the modifying layer of the PSDC electrolyte with partial *p*-type electron conductivity was deposited on the cathode side to reduce the polarization resistance. 

The method of electrophoretic deposition was applied to form thin electrolyte layers on a dense supporting membrane. The electrical conductivity of the dense SDC membrane surface was achieved by the synthesis of a conductive polypyrrole sublayer. The kinetic parameters of the EPD process from the PSDC suspension were studied. The volt-ampere characteristics and power output of the obtained SOFC cells with the BCS-CuO anode blocking layer and with/without the PSDC modifying layer on the cathode side were investigated. The effect of increasing the power output and OCV of the cell with the PSDC cathode sublayer electrolyte membrane by decreasing the ohmic and polarization resistance of the cell was demonstrated. The specific power of the cell with both the BCS-CuO anode barrier layer and the PSDC cathode layer reached 159, 240, 326, and 419 mW cm^−2^ at temperatures of 650, 700, 750, and 800 °C, respectively. These characteristics were significantly higher than those obtained for the cell without PSDC, and they exceeded the performance of the cell with a supporting SDC electrolyte of the similar thickness (500 μm) with BaCe_1−x_Sm_x_O_3−δ_ anode barrier layer and with a Sm_0.5_Sr_0.5_CoO_3_ cathode, presented in the literature. Moreover, the introduction of the PSDC layer resulted in increasing the OCV value, while the thin BCS-CuO layer deposited on the thick SDC electrolyte demonstrated poor protective properties. 

The use of NiO-BCGCu cermet anode and LSFC-SDC/LNF-EDB-CuO multilayer ceramic cathode was found to be more efficient than Pt-activated electrodes. The PSDC layer also had a significant depolarizing effect on the polarization resistance of the cathode, reducing the total *R_p_* value down to 0.5, 0.2, 0.1, and 0.05 Ohm cm^2^ at temperatures of 650, 700, 750, and 800 °C, respectively. However, an efficient anode design is still required to further enhance the SOFC performance. 

The developed approach of applying of the anode barrier layer in combination with a modifying cathode layer can be recognized as an effective solution for enhancing the performance of IT-SOFCs with a supporting MIEC electrolyte membrane. In addition, this approach can be further developed for the use in SOFCs with a three-layer thin film electrolyte. 

## Figures and Tables

**Table 1:** Characteristics of the electrode and electrolyte materials.

Material (Application)	Synthesis Method	Crystal Lattice Type, Space Group	Lattice Parameters, Å	Specific Surface Area, m^2^ g^−1^
SDC (supporting membrane)	SSR	cubic type structure, Fm-3m	a = 5.4324 (3)	2.5 (1)
PSDC	CNC	cubic type structure, Fm-3m	a = 5.4314 (3)	15.0 (3)
BCS-CuO	CNC	orthorombic structure, Pnma	a = 6.2311 (1), b = 8.8013 (2), c = 6.2272 (1)	3.0 (1)
BCGCu	SSR	orthorombic structure, Pmcn	a = 6.2523 (1), b = 8.7912 (2), c = 6.2180 (1)	2.4 (1)
EDB	CNC	cubic type structure, Fm-3m	a = 5.45082 (3)	1.6 (1)
LSFC	CNC	rhombohedral structure, R3C	a = 5.4717 (2), b = 5.4717 (2), c = 13.4505 (4)	2.2 (1)
LNF	MP	rhombohedral structure, R3C	a = 5.5220 (2), b = 5.5220 (2), c = 13.3028 (4)	5.5 (1)

**Table 2:** Electrokinetic properties of the suspensions (10 g/L) of the PSDC powder and the BCS-CuO powder (with/without iodine).

Suspension	UT, min	Zeta Potential, mV (pH)
PSDC	5 125	+16 (5.5) +23 (6.4)
BCS-CuO BCS-CuO + iodine	5 125 125	+11 (5.1) +11 (4.2) +11 (3.7)

**Table 3:** Characteristics of single SOFCs with supporting and thin-film SDC-based electrolyte membranes.

Electrochemical Cell, Layer Thickness, μm	OCV, mV/T, °C	SPD, mW cm^−2^/T, °C	Ref.
NiO-BCGCu (40)/ BCS-CuO (18)/SDC (550)/ LSFC-SDC (30)/ LNF-EDB-CuO (40) (EPD)	805 (650) 789 (700) 760 (750) 712 (800)	90 (650) 119 (700) 159 (750) 186 (800)	This study
NiO-BCGCu (40)/ BCS-CuO (18)/SDC (550)/ PSDC (6)/LSFC-SDC (30)/ LNF-EDB-CuO (40) (EPD)	918 (650) 872 (700) 827 (750) 782 (800)	159 (650) 240 (700) 326 (750) 419 (800)	This study
NiO-SDC/BCS (13)/	1002 (600)	76 (600) 170 (700)	[18]
SDC (500)/Sm_0.5_Sr_0.5_CoO_3_	857 (900)	278 (800) 399(900)	
NiO-SDC/ SDC (500)/Sm_0.5_Sr_0.5_CoO_3_	964 (600) 888 (700) 823 (800) 715 (900)	62 (600) 126 (700) 170 (800) 85 (900)	[17]
NiO-SDC/Ce_0.9_Sm_0.1_O_1.95_ (500)/ La_0.6_Sr_0.4_Fe_0.8_Co_0.2_O_3_	838 (600) 768 (700) 714 (750)	38 (600) 78 (700) 104 (750)	[35]
NiO-SDC/Ce_0.9_Sm_0.08_Pr_0.02_O_1.95_ (500)/La_0.6_Sr_0.4_Fe_0.8_Co_0.2_O_3_	783 (600) 751 (700) 717 (750)	49 (600) 103 (700) 126 (750)	[35]
NiO-BCS-CuO (800)/BCS-CuO (13)/ SDC (18)/Pt (30) (EPD)	1050 (650) 1000 (700) 900 (750)	50 (650) 80 (700) 160 (750)	[28]
NiO-SDC/SDC (30)/La_0.6_Sr_0.4_CoO_3−δ_ (EPD)	~700 (600)	161 (500) 281 (600) 272 (700)	[68]
NiO-BCS-SDC/BCS-SDC (30)/ Sm_0.5_Sr_0.5_ CoO_3−δ_–Ce_0.8_ Sm_0.2_O_1.9_	1017 (600) 1001 (650) 976 (700)	216 (600) 343 (650) 505 (700)	[49]
NiO-BCS/SDC modified TiO_2_ (30)/Pt (EPD)	920 (750)	125 (750)	[67]

## Data Availability

Not applicable.

## Supplementary Materials

The following supporting information can be downloaded at: https://www.mdpi.com/article/10.3390/membranes13050484/s1, Figure S1: XRD patterns obtained for the powders after the final calcination/sintering step: (a) SDC powder (1600 °C); (b) PSDC powder (800 °C); (c) BCS-CuO powder (1150 °C); (d) BCGCu powder (1100 °C); (e) EDB powder (600 °C); (f) LSFC powder (1100 °C); (g) LNF powder (900 °C). Figure S2: Electrode response obtained under OCV conditions in the SOFC mode for the single cells with the Ni-BCGCu anode and the LSFC-SDC/LNF-EDB-CuO cathode and the supporting electrolyte membrane comprising with the BCS-CuO anode layer (18 μm)—(a); with the BCS-CuO anode layer (18 μm) and the PSDC cathode layer (6 μm); Figure S3: Cross-sectional images for the Ni-BCGCu/BCS-CuO/SDC/PSDC/LSFC-SDC/LNF-EDB-CuO cell (SDC2 cell) after testing in the SOFC mode: SEM images obtained on the cathode side—(a–c); SEM images on the anode side—(d–f) Cross-sectional SEM images, ×1500 magnification—(a,d); integrated maps of element distribution—(b,e); individual maps of elements—(c,f).
