# Supplementary material for: Performance Enhancement of Ce0.8Sm0.2O1.9-Supported SOFC by Electrophoretic Formation of Modifying BaCe0.8Sm0.2O3 and Ce0.8Sm0.1Pr0.1O1.9 Layers
Source: Membranes (Basel). 2023 Apr 29;13(5):484. doi: 10.3390/membranes13050484 (PMC10222043; doi:10.3390/membranes13050484)
Supplement: Supplementary file 1 [file membranes-13-00484-s001.zip › membranes-2327474-supplementary.pdf]

Supplementary materials

Performance enhancement of $Ce_{0.8}Sm_{0.2}O_{1.9}$-supported SOFC by Electrophoretic Formation of Modifying $BaCe_{0.8}Sm_{0.2}O_3$ and $Ce_{0.8}Sm_{0.1}Pr_{0.1}O_{1.9}$ Layers

Elena Pikalova[1,2], Elena Kalinina[3,4*]

[1] Laboratory of Solid Oxide Fuel Cells, Institute of High Temperature Electrochemistry, Ural Branch of the Russian Academy of Sciences, Yekaterinburg 620137, Russia; e.pikalova@list.ru
[2] Department of Environmental Economics, Graduate School of Economics and Management, Ural Federal University, Yekaterinburg 620002, Russia; e.pikalova@list.ru
[3] Laboratory of Complex Electrophysic Investigations, Institute of Electrophysics, Ural Branch of the Russian Academy of Sciences, Yekaterinburg 620016, Russia; jelen456@yandex.ru
[4] Department of Physical and Inorganic Chemistry, Institute of Natural Sciences and Mathematics, Ural Federal University, Yekaterinburg 620002, Russia; jelen456@yandex.ru
* Correspondence: jelen456@yandex.ru (E.K.); Tel.: +7-343-267-87-82 (E.K.)

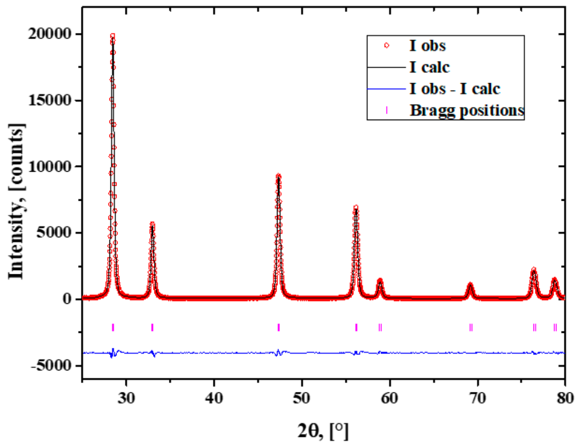

(a) SDC

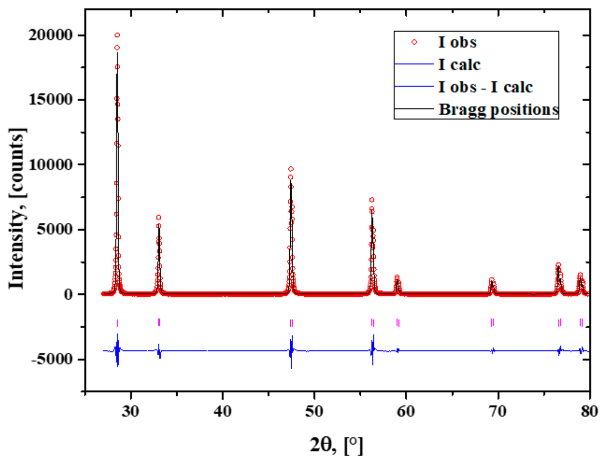

(b) PSDC

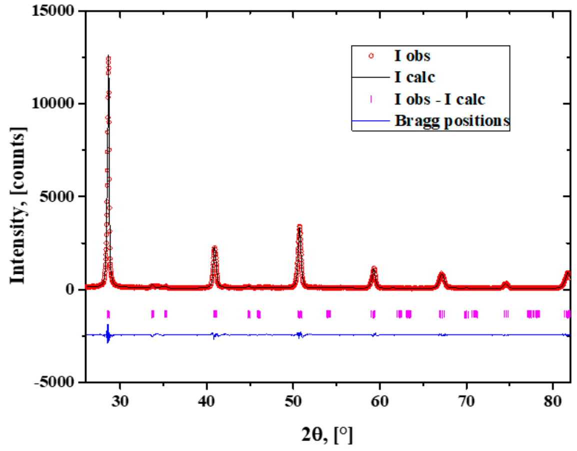

(c) BCS-CuO

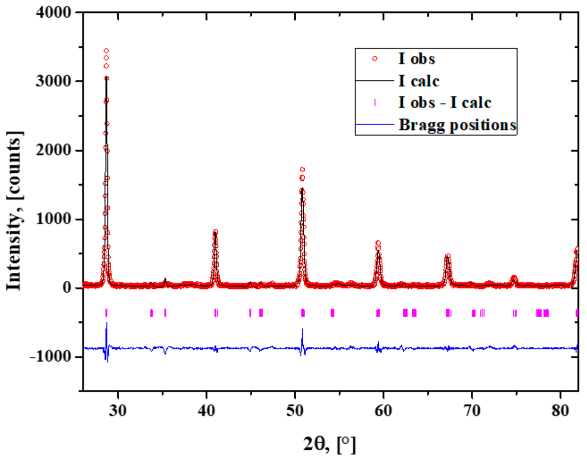

(d) BCGCu

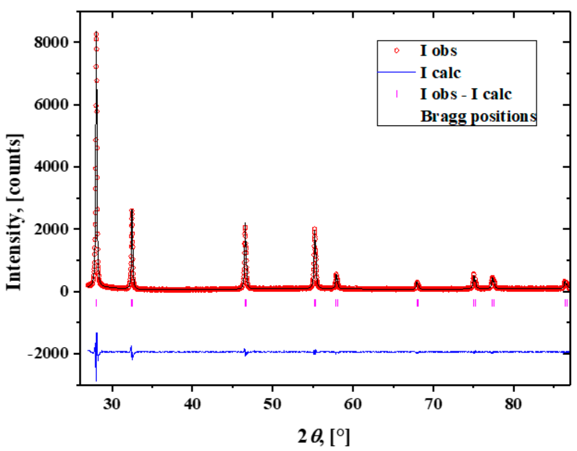

(e) EDB

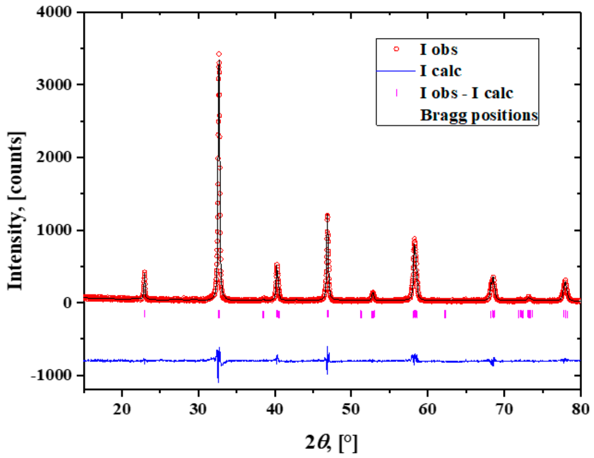

(f) LSFC

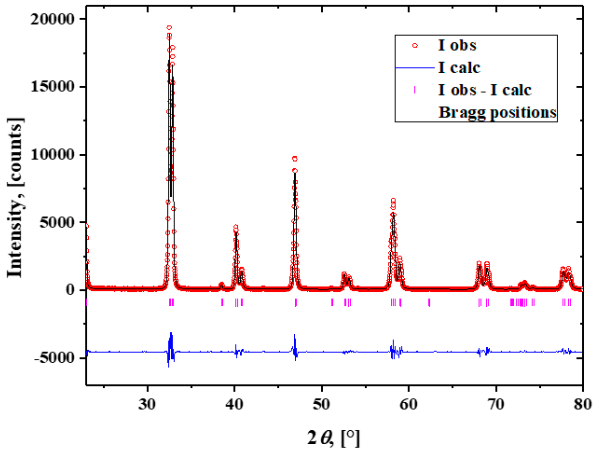

(g) LNF

Figure S1: XRD patterns obtained for the powders after the final calcination/sintering step: (a) SDC powder (1600 °C); (b) PSDC powder (800 °C); (c) BCS-CuO powder (1150 °C); (d) BCGCu powder (1100 °C); (e) EDB powder (600 °C); (f) LSFC powder (1100 °C); (g) LNF powder (900 °C).

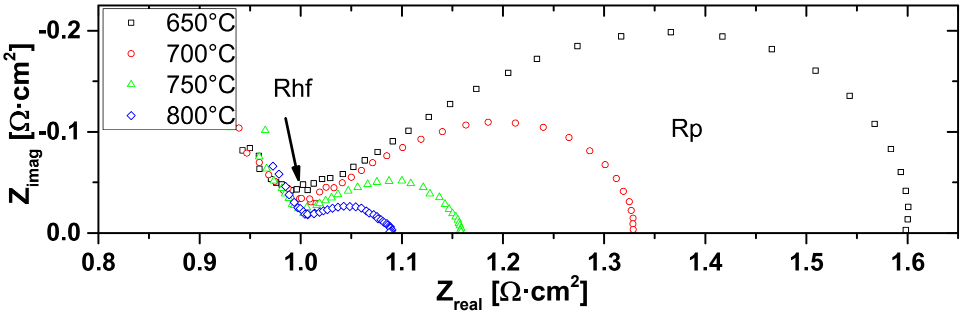

(a)

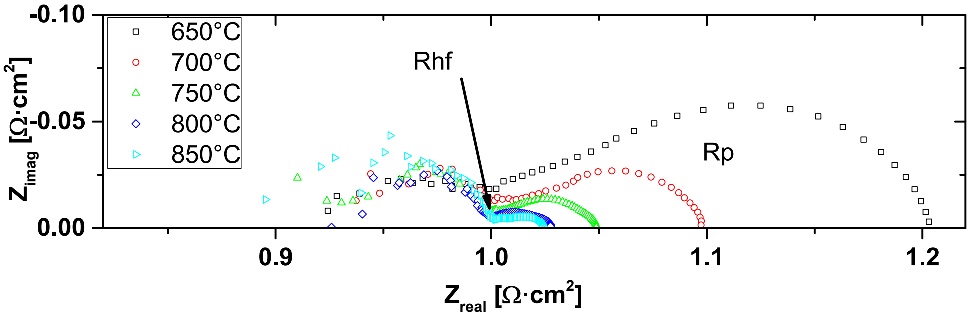

(b)

Figure S2 Electrode response obtained under OCV conditions in the SOFC mode for the single cells with the Ni-BCGCu anode and the LSFC-SDC/LNF-EDB-CuO cathode and the supporting electrolyte membrane comprising with the BCS-CuO anode layer (18 μm) – (a); with the BCS-CuO anode layer (18 μm) and the PSDC cathode layer (6 μm)

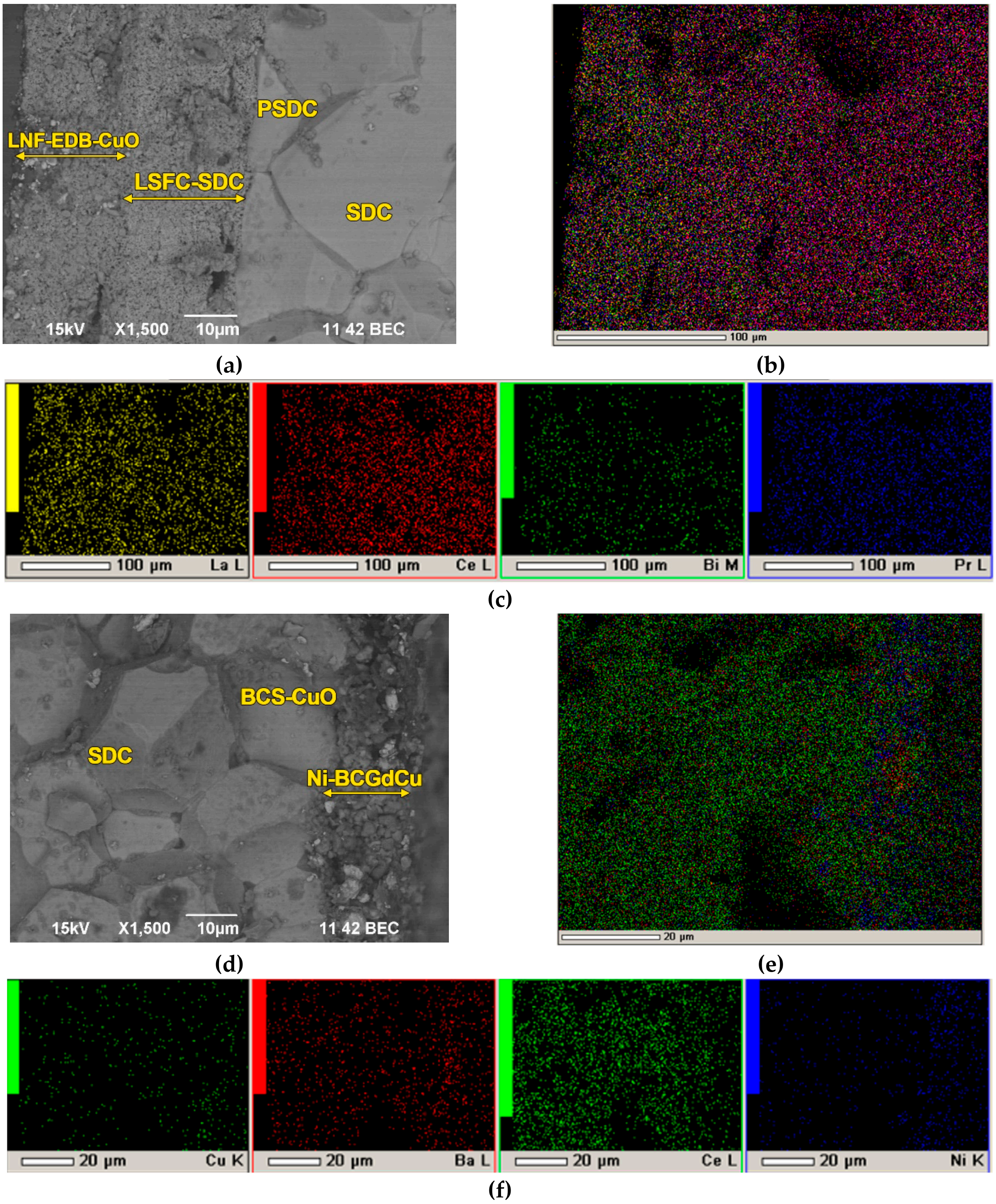

Figure S3 Cross sectional images for the Ni-BCGCu/BCS-CuO/SDC/PSDC/LSFC-SDC/LNF-EDB-CuO cell (SDC2 cell) after testing in the SOFC mode: SEM images obtained on the cathode side - (a), (b), (c); SEM images on the anode side - (d), (e), (f)

Cross sectional SEM images, x1500 magnification - (a), (d); integrated maps of element distribution - (b), (e); individual maps of elements - (c), (f)
